# Variation in Seed Morphological Traits Affects the Dispersal Strategies of *Chromolaena odorata* Following Invasion

**DOI:** 10.3390/plants13131747

**Published:** 2024-06-24

**Authors:** Yangping Li, Guofen Wang, Yupeng Geng, Ju Li, Yulong Feng

**Affiliations:** 1 CAS Key Laboratory of Tropical Forest Ecology, Xishuangbanna Tropical Botanical Garden, Chinese Academy of Sciences, Mengla 666303, China; 2 Environment and Plant Protection Institute, Chinese Academy of Tropical Agricultural Sciences, Haikou 571101, China; guofenwang7810@163.com; 3 Yunnan Key Laboratory of Plant Reproductive Adaptation and Evolutionary Ecology, School of Ecology and Environmental Sciences, Yunnan University, Kunming 650500, China; ypgeng@ynu.edu.cn; 4 Public Technology Service Center, Xishuangbanna Tropical Botanical Garden, Chinese Academy of Sciences, Mengla 666303, China; liju@xtbg.ac.cn; 5 Liaoning Key Laboratory for Biological Invasions and Global Changes, College of Bioscience and Biotechnology, Shenyang Agricultural University, Shenyang 110866, China; yl_feng@tom.com

**Keywords:** *Chromolaena odorata*, functional traits, seeds, dispersal, seed mass, seed germination, native range, introduced range

## Abstract

Seed germination and dispersal have an important impact on the establishment and spread of invasive plants. Understanding the extent of intraspecific seed trait variations can enhance our understanding of how invasive plants respond to environmental change after introduction and help predict the dynamic of invasive species under future environmental conditions. However, less attention has been given to the variation in seed traits within species as opposed to among species. We compared seed production, seed morphological traits, dispersal ability, and seedling performance of *Chromolaena odorata* from 10 introduced populations in Asia and 12 native populations in America in a common garden. The results showed that range (introduced vs. native) and climate affected these traits. Compared with the native population, the introduced populations had higher seed numbers per capitula, lighter seeds, and higher potential dispersal ability seeds (lower terminal velocity) but lower germination rates and seedling lengths. Climatic clines in seed numbers per capitula and pappus length were observed; however, the clines in pappus length differed between the introduced and native populations. Trait covariation patterns were also different between both ranges. In the native populations, there was a trade-off between seed numbers per capitula and seed mass, while this relationship was not found for the introduced populations. These results indicate that *C. odorata* alters the ecological strategy of seed following invasion, which facilitates its establishment and fast dispersal and contributes to successful invasion in the introduced ranges.

## 1. Introduction

Intraspecific variation in functional traits across environmental gradients represents an important driver of plant performance and reflects the adaptations of species to environmental conditions [1]. Seeds serve as crucial reproductive units in higher plants, and the traits associated with their germination and dispersal processes significantly influence plant performance, spread, population establishment, and dynamics, ultimately shaping their evolutionary trajectory [2,3,4,5]. Understanding seed variation distributed across different organization levels (i.e., individual, population, and space) and interactions with each other is helpful for understanding the evolution of seeds traits; however, previous studies have predominantly focused on interspecific comparisons [6]. Invasive species with a wide distribution provide an excellent model for investigating intraspecific variations in seed traits under diverse environmental conditions. Such studies can enhance our understanding of how plants respond to environmental changes, thereby facilitating the prediction of invasive species dynamics under future environmental conditions.

Among the various seed types, wind-dispersed seeds have been extensively investigated [7,8]. The interrelationships among traits such as seed mass, pappus area, and dispersal ability have been thoroughly assessed in numerous species [6,7]. Seed mass is associated with a diverse range of functions [9]. Previous studies have documented that larger or heavier seeds exhibit reduced dispersal potential but enhanced emergence capabilities and superior performance during the early establishment stages [10,11,12]. The trade-off between seed mass and the number of seeds per plant has been widely established across numerous species [13]. Furthermore, in the case of plumed seeds, dispersal ability is typically linked to the pappus structure [2,14,15]. The terminal velocity of seeds was positively correlated with the square root of wing loading (the ratio of mass to pappus area) [16]. Chen and Giladi (2020) also demonstrated a significant negative correlation between the pappus width/opening angle of *Geropogon hybridus* (L.) Sch. Bip. (Asteraceae), an annual wind-dispersed plant species, diaspore terminal velocity, and seedling emergence [2]. These studies provided valuable insights into the variation patterns and covariation relationships among seed traits under different environmental conditions. However, limited attention has been paid to examining this relationship within species. 

Invasive plants are introduced into novel and unusual environments, where their seeds experience distinct and intense selection pressures compared with their native range [17,18,19,20]. Different biotic environments (e.g., release from specialist enemies present in the native range) [17,21] drive modifications in functional traits (e.g., seed traits), resulting in changes in seed germination and dispersal potential [6,22,23]. For example, Liu et al. (2020) reported that *Spartina alterniflora* Loisel. (Poaceae) from introduced ranges produced four times more seeds than conspecifics from their native ranges in both the field and the common garden [24]. Other studies have indicated that seeds from introduced populations germinated better or have different germination strategies than those from native populations [20,25,26]. Huang et al. (2015) found that seed dispersal potential (plume loading of seeds) significantly decreased with an increasing distance from the source population under field conditions, suggesting variation in spreading ability following invasion [3]. Furthermore, following introduction, invasive species experience different climatic conditions that may induce rapid post-introduction evolution, leading to changes in functional traits [27,28,29]. Rosche et al. (2019) indicated that among-population variation in the performance of the invasive species *Erigeron canadensis* (also known as *Conyza canadensis*) (L.) Cronquist (Asteraceae) is primarily determined by the climatic conditions within each range [30]. In terms of seed traits, Liu et al. (2020) found a latitudinal cline for *S. alterniflora* in the introduced but not native range [24]. Additionally, it should be noted that the pattern of covariation in functional traits may differ between introduced and native ranges. Different trait coordinates have been observed for leaf functional traits in some species [28,31,32]. For instance, Tewes and Müller (2018) found that native, introduced, and naturalized populations showed different trait coordinates [33]. Nevertheless, there is limited knowledge regarding the variations and covariations among seed traits, as well as their impact on seed germination, dispersal potential, and seedling performance during biological invasions.

*Chromolaena odorata* (L.) R. M. R.M. King & H. Rob. (Asteraceae) is native to America, a very widely distributed tropical shrub in Asia, Oceania, and Africa, and considered one of the world’s worst weeds [34]. Once established, it can form monoculture stands, and successfully invade diverse habitats with a wide range of environmental gradients, such as cultivated lands, abandoned fields, forest gaps, wastelands, forest trails, fence rows, roadsides, and forest margins [35,36]. It flowers seasonally, predominantly during the dry season. Reproduction is often apomictic in nature, although insects of various types visit flowers [37]. Each head consists of 15–35 tiny flowers arranged in corymbs, and there are 13–40 seeds tiny flowers. It can produce abundant quantities of seeds, with estimates ranging from 2000 (<1 year) to 260,000 (10 years) seeds per square meter [38]. A significant proportion of seeds are deposited into the soil, contributing to the formation of a seed bank [39]. These seeds have been observed to remain viable for up to six years in the soil [40]. The seeds (achenes) were topped with a pappus and dispersed away from their original location through wind or by adhering to fur, feathers, and clothing [41]. Additionally, small seeds can disperse by attaching directly to vehicles or becoming embedded in mud and subsequently picked up by vehicles [42,43], as well as being facilitated by the airflow generated by vehicle movement [44]. Blackmore (1998) demonstrated that the dispersal distance of *C. odorata* seeds is enhanced in the areas that are crossed by vehicles [45]. The germination process is facilitated by humidity and temperatures exceeding 20 °C [41]. Biogeographical studies have indicated that the introduced population of *C. odorata* displays fast growth economic traits, high physical defense, and drought escape strategies relative to the native populations [28]. In terms of seed traits, variations in seed germination among populations have been observed [46]. However, it remains unclear how seed traits vary and covary between introduced and native species across a wide geographic ranges.

In this study, we compared the seed production, seed traits, dispersal ability, and seedling performance of *C. odorata* from 10 introduced populations in Asia and 12 native populations in Central and South America. The following questions were addressed: (1) Are there biogeographical differences in these traits between the introduced and native ranges? (2) Do seed traits vary with climatic gradients? (3) Are there any correlations between seed size, pappus traits, and seed germination/dispersal characteristics?

## 2. Results

### 2.1. Seed Trait Variation

The morphological traits of the seeds varied substantially. Seed mass ranged from 0.13 to 0.60 mg, pappus length varied between 0.19 and 0.58 cm, and the number of seeds per capitula ranged from 13 to 40 (Table S2). The distribution of variance in seed mass was well balanced across the three organizational levels (Figure S2). Both the range and population levels contributed equally to explaining the variance in the number of seeds per capitula, whereas the majority of the variance in pappus length was observed at the population and plant levels (Figure S2).

Seed dispersal and seedling emergence traits exhibited considerable variation. Notably, the length of seedlings showed a 20-fold range (ranging from 3.6 to 69.0 cm), while the germination rate displayed a 12-fold range (ranging from 6.0 to 78.0%). Additionally, the terminal velocity ranged between 32.2 and 112.3 m s^−1^ (Table S2). The distribution of variance in these traits was well-balanced across the three levels of organization (Figure S2).

### 2.2. Effect of Range on Seed Traits

**Figure 1:**
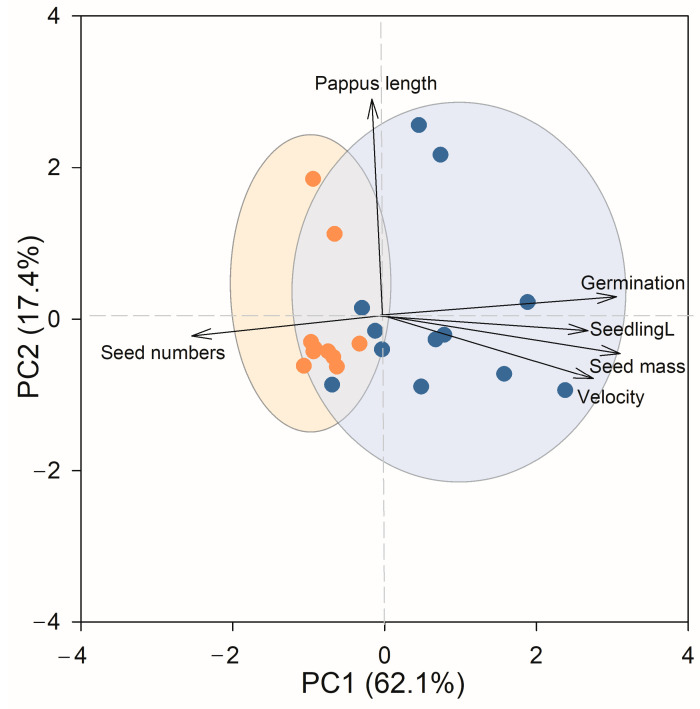
Biplot of principal component analysis (PCA) for the six traits of 10 introduced (circles in orange) and 12 native (circles in blue) populations of *Chromolaena odorata* grown. Germination, germination rate; seedlingL, seedling length; velocity, terminal velocity; seed numbers, seed number per capitula.

The principal component analysis (PCA) accounted for 79.5% of the total variation in seed traits. In particular, the first and second axes explained a substantial amount of the variation (PC1 = 62.1%; PC2 = 17.4%). The first principal component exhibited strong correlations with the seed number per capitula, seed mass, seed germination rate, terminal velocity, and seedling length. Conversely, the second principal component was primarily associated with pappus length (Figure 1). Geographical regions played a significant role in sample clustering within the ordination space, demonstrating separation between the introduced and the native ranges along the first axes (T-test result of PC1 scores: *t* = 5.657, *p* < 0.001) (Figure 1). However, there was overlap between the native and introduced ranges along the second axis (T-test result of PC2 scores: *t* = 0.170, *p* = 0.867) (Figure 1).

**Figure 2:**
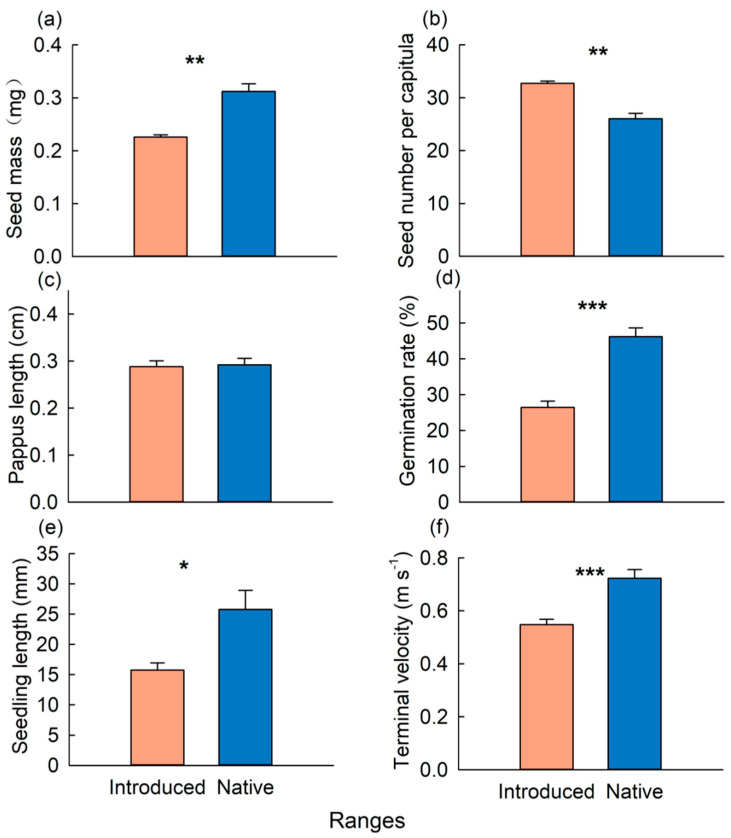
Seed mass (**a**), seed number per capitula (**b**), pappus length (**c**), germination rate (**d**), seedling length (**e**), and terminal velocity (**f**) of *Chromolaena odorata* from the the introduced and native populations. *, **, and *** indicate *p* < 0.05, *p* < 0.01, and *p* < 0.001.

The origin of the seeds (introduced and native ranges) significantly influenced four out of the six seed traits (Table 1). The introduced populations exhibited a higher seeds number per capitula (32%) than the native populations, whereas the former had a significantly lower seed mass (31%) than the latter (Figure 2, Table 1 and Table S3). There were no significant differences in pappus lengths between the introduced and native ranges (Figure 2). The introduced population of *C. odorata* displayed a lower germination rate (45%), shorter seedling length (39%), and slower terminal velocity (24%) than the native population (Figure 2, Table 1 and Table S3).

### 2.3. Effect of Climatic on Seed Trait 

**Figure 3:**
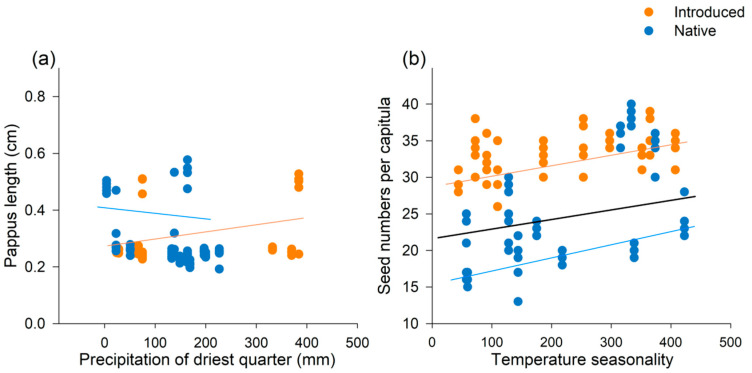
Relationships between climatic conditions vs. seed traits for the 12 native and 10 introduced populations of *Chromolaena odorata*. (**a**) Relationship between pappus length and precipitation of driest quarter. (**b**) Relationship between seed number per capitula and temperature seasonality. Orange dots and lines indicate data from introduced populations and blue dots and lines indicate data from native populations. The black line indicates data that includes both range populations. Data are presented at the individual level. Lines are shown for models that had significant relationships at *p* < 0.05.

Variations in the two seed traits was observed across climatic gradients (Table 1). With increasing temperature seasonality, there was a corresponding increase in the number of seeds per capitula (Figure 3). Additionally, the precipitation of the driest quarter affected pappus length; however, these effects differed between the introduced and native populations (see Table 1 for a significant interaction between range and climate). Among the native populations, there was a negative correlation between seed and pappus length with increasing precipitation of driest quarter, whereas an opposite trend was observed among the introduced populations (Figure 3).

### 2.4. Seed Traits Relationship

**Figure 4:**
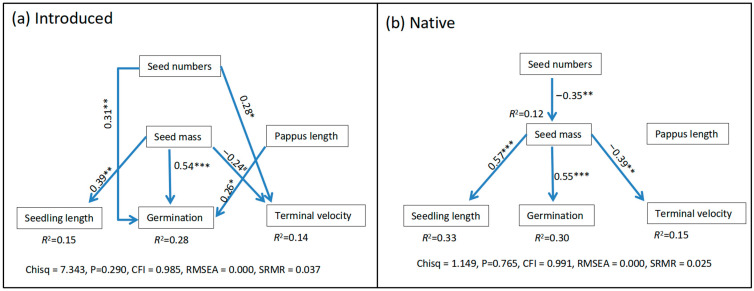
Structural equation model (SEM) of the effects of seed trait (seed numbers per capitula, seed mass and pappus length) on germination, terminal velocity, and seedling length for the introduced (**a**) and native populations (**b**). *R*^2^ values associated with response variables indicate the percentage of variation explained by the explanatory variables. Values associated with solid arrows represent standardized path coefficients. ^#^, *, **, and *** indicate significant effects at *p* < 0.10, *p* < 0.05, *p* < 0.01, and *p* < 0.001 levels.

Seed mass exhibited a significant positive correlation with germination rate and seedling length for both introduced and native populations (Figure 4). However, there were differences in the trait covariation between the introduced and native populations. For native populations, there was a trade-off between seed numbers per capitula and seed mass, as well as between seed mass and terminal velocity; however, these trade-off relationships were not observed in the introduced populations (Figure 4). In the introduced populations, there was a positive relationship between the seed numbers per capitula and the germination rates and terminal velocity. In contrast, this relationship was not observed in the native populations (Figure 4).

## 3. Materials and Methods

We collected seeds of *C. odorata* in March 2010 from 12 populations in America and 10 populations in the introduced range in Asia (Table 2). Sampled populations were located at least 100 km apart from one another. Within each population, seeds were collected from 10 to 15 randomly chosen plants at least 10 m apart from one another. These seeds of all populations were germinated and grown in July 2010 in a common garden, Xishuangbanna Tropical Botanical Garden (XTBG) (21°56′ N, 101°15′ E), Chinese Academy of Science, Yunnan Province, Southwest China. To avoid cross pollination between populations by insects, we covered the capitula, which were ready for seed collection, with a nylon bag with hole diameter of 0.18 mm during the flowering phase. From December 2015 to February 2016, seeds were collected again from 10 to 12 individuals per population.

The climate variables of each sampling site were obtained from WorldClim, including the temperature seasonality and the precipitation of driest quarter (downloaded from http://worldclim.org/version2 (accessed on 2 February 2019); Harris et al., 2014 [47]; Table S1).

Five plants were selected from each population, and 20 capitula were chosen from each plant to count the seed numbers per capitula. In total, 2200 capitula were collected (20 flower heads × 5 plants × 22 populations). The average value for each plant was calculated as a replicate. To measure other traits of the seeds, we collected three sets of 50 seeds from each plant and selected five plants per population. Pappus length was measured by scanning each sample using an EPSON flatbed scanner (v550; Epson China, Beijing, China) and analyzing the images with ImageJ version 1.25a [48] (see Figure S1 for measurement details). Each sample of 50 seeds was weighed using an analytical balance (accuracy = 0.0001 g), and the mass of one seed was calculated. The average value for each plant was calculated as a replicate.

To estimate the potential dispersal velocity, three samples were selected from each of the five plants, each consisting of 20 seeds with a fully expanded pappus. The subsidence time of each seed was measured by dropping it into a transparent plastic cylinder (1 m height and 10 cm radius) at ambient temperature, and the terminal velocity was calculated. Prior to the measurement, deionizing fans (SL-001, Shilaide anti-static equipment Co., Ltd., Dongguan, China) were employed to treat the plastic cylinder and eliminate any influence of static electricity on the hands of the operating personnel. The average value for each plant was calculated as a replicate.

To evaluate germination, three samples of 50 seeds per plant were combined and germinated on sand in Petri dishes at a temperature of 25 °C, under a 12 h photoperiod (light intensity of 250 μmol m^−2^ s^−1^). After two weeks, we recorded the number of germinated seeds to calculate the germination rate and measured the length of the seedlings. 

### Statistical Analysis

Variations in seed traits were assessed across three hierarchical levels of organization: (1) between introduced and native ranges, (2) among populations, and (3) within individual plants of a population. To estimate the partitioning of variance for the seed traits at these three levels, variance component analyses were conducted using general linear models. Each model included the response variable as one of the traits, with range, population, and plant considered as random effects. The models were fitted using the restricted maximum likelihood method implemented in the R package lme4 1.1-17 [49].

We conducted a principal component analysis (PCA) to analyze the associations between seed traits in the sampled populations using population mean trait values by using IBM SPSS Statistics for Windows 25.0 (Armonk, NY, USA: IBM Corp.). Then, to identify minimal adequate models, we removed nonsignificant fixed effects in a stepwise backward manner if *p* > 0.05, based on the likelihood ratio test using IBM SPSS Statistics for Windows. We used generalized linear mixed models to test the effect of range and climate (temperature seasonality) on seed numbers per capitula (Poisson response) with population nested range as random factors using the glmmTMB package in R [50]. To consider the demographic history in the patterns of seed trait differences in the mixed models, we added population mean STRUCTURE *q*-scores as a random effect. We used the Bayesian clustering method STRUCTURE v2.3.4 [51] to calculate *q*-scores for the most likely number of clusters K (=2) using microsatellite (SSR) data from the previous study of Li et al. (2020) [52]. For other variables, we fitted a linear mixed model to test the effect of range (invasive vs. native) and climate on populations, with range and *q*^2^ as random factors nested within populations, using individual means as replicates in the R package lme4 1.1-17 [49].

Structural equation modeling (SEM) was used to detect the effects of seed morphological traits on germination and terminal velocity in introduced and native populations using the lavaan package in R [53]. We used criteria including *p* values, chi-square values, comparative fit index (CFI), and root mean square error of approximation (RMSEA) to evaluate the SEM fit. The fit was considered perfect when the chi-square test was insignificant (*p* > 0.05), CFI was close to 1.000, and RMSEA and SRMR values were lower than 0.05 [54].

## 4. Discussion

Seed traits are thought to undergo strong environmental selection because of their significant implications for fitness [2,55]. Following their introduction, selection pressures from novel abiotic and biotic environmental conditions in the introduced ranges may lead to significant changes in the seed traits of invasive species. However, few biogeographical studies have focused on these seed traits. Our study revealed significant variations in the seed and seedling traits of *C. odorata* at both the range and population levels, providing this invasive species with a broad dispersal capacity and the potential for seedling emergence across diverse environmental gradients, as well as distinct seed strategies between the introduced and native ranges. The distribution of variance in seed morphological traits and numbers spans all organizational levels, indicating that processes operating at the range, population, and individual plant levels collectively contribute to determining these traits.

### 4.1. Seed Trait Divergence between Introduced and Native Populations

Invasive plants may allocate resources for growth and reproduction due to their release from native specialist enemies, as proposed by the evolution of the increased competitive ability hypothesis [21,56]. Invasive plants also exhibit increased seed production. For example, Correia et al. (2016) found that two invasive Australian woody legumes, *Acacia dealbata* Link (Fabaceae) and *A. longifolia* (Andrews) Wild., had higher seed production in their invaded ranges than in their native ranges, which was attributed to escape pre-dispersal predation in the introduced ranges, and more resource allocation to reproduction [17]. Similarly, our study found more seeds per capitula in the introduced ranges than in the native ranges. However, the introduced populations also had a lower seed mass, indicating a trade-off between seed mass and number. These findings are inconsistent with those reported by Correia et al. (2016), who observed no trade-offs between seed mass and production [17]. Despite the lower seed mass in the introduced populations, these seeds exhibited higher dispersal potential, characterized by a lower terminal velocity. Field studies have found that wind-dispersed seeds of *C. odorata* contribute to short-distance dispersal (>80 m), while long-distance dispersal relies mainly on vehicles [45]. However, these changes in seeds within the introduced population enable them to colonize more ecological niches surrounding their habitat and prevent other species from establishing themselves there, thereby enhancing their establishment and spread. Another study on the wind-dispersed seeds of the invasive species *Mikania micrantha* Kunth (Asteracea) showed increased dispersal ability with increasing distance from the source population in their introduced range [3]. These findings suggest that enhanced seed-dispersal ability, even over short distances, plays an important role in facilitating biological invasions. 

Seed mass is a crucial trait that affects germination and seedling performance. In the present study, we observed a lower germination rate in introduced populations with lighter seeds. Some previous studies have reported heavier seeds in the introduced range compared to native ranges [57,58,59], suggesting that an increase in seed size leads to higher offspring growth [17,60]. Some studies have been conducted on wind-dispersed seeds, where lighter seeds disperse better but may have lower emergence potential and competitive advantage in early establishment stages [10,11,12]. Nevertheless, the fact that *C. odorata* has lighter seeds does not necessarily indicate lower competitiveness. A previous study by Li et al. [52] (2020) found that *C. odorata* exhibited lower height and biomass in low-nutrient environments in the introduced ranges but still displayed a higher competitive ability (lower percent change in competitive treatment) [52].

### 4.2. Seed Trait Variation across Climatic Gradient

Seed traits may exhibit variation in response to diverse climatic conditions. In the present study, we observed that populations inhabiting sites characterized by high-temperature seasonality displayed higher seed numbers per capitula compared to those from sites with low-temperature seasonality, indicating an adaptive strategy for coping with temperature fluctuations through increased seed production. Distinct clines were observed between the introduced and native populations. The introduced populations from locations experiencing greater precipitation during the driest quarter exhibited longer pappi, whereas the native populations demonstrated the opposite trend. Pappus length is a crucial dispersal structure and elongation of the pappus may enhance plume loading [16]. Notably, *C. odorata* seeds reached maturity during the dry season. In habitats with higher levels of moisture (characterized by elevated precipitation in the driest quarter), introduced populations may have adapted to these conditions by augmenting their pappus length to improve dispersal ability.

On the contrary, native populations showed contrasting responses to precipitation conditions. These results indicate that climate primarily acts as a selective pressure driving variation in pappus traits among populations and explains for the similar pappus lengths observed between the introduced and native populations. Regarding the aboveground traits of *C. odorata*, distinct clines across climatic gradients have been identified between the introduced and native ranges [28]. These findings indicate that climatic niches following introduction also contributed to the spread of *C. odorata* in the introduced ranges under varying climatic conditions.

### 4.3. Seed Traits Covariation

The dispersal ability of plumed seeds is typically associated with the seed size and pappus structure [2,7]. Previous studies indicated a close correlation between the terminal velocity of plumed seeds and the square root of plume loading (the ratio of the pappus area to seed mass) [16]. However, our study found that the terminal velocity of *C. odorata* seeds was correlated with seed mass rather than with the pappus traits (length). This result is not consistent with an intraspecific study on *G. hybridus* [2,61], where the dimensions of pappus (width and length) accounted for a greater proportion of the variation in seed terminal velocity compared to the measurements of mass (diaspore, pappus, or achene) on terminal velocity. In *C. odorata*, the adjustment of pappus structure contributed little to seed dispersal ability compared to seed mass.

The different abiotic and biotic environments in introduced ranges not only lead to variations in traits, but also result in distinct patterns of trait coordination [24,28,62]. In the present study, we observed strong correlations among these seed traits; however, the patterns differed between the introduced and native ranges. For instance, higher germination and seedling length were associated with greater seed mass in both ranges; however, we also found a positive correlation between pappus length and germination in the introduced range. Similar findings were reported by Chen and Giladi’s (2020), who showed that *G. hybridus* pappus traits were correlated with seedling emergence within dispersive diaspores [2]. Nevertheless, the morphological traits of seed pappi are not reliable predictors of germination because various factors can influence the process [2]. If diaspore traits indeed affect germination, their effects are likely integrated to form a complex relationship between dispersal and post-dispersal functions [2]. Additionally, we observed a significant positive relationship between seed number per capitula and germination in the introduced ranges but not in the native ranges. Such trait relationships in the introduced ranges might arise from decoupling the relationships between seed number and mass in the introduced range. However, it is plausible that this could be attributed to the limitations of the sampling method. Our study focused solely on quantifying the number of seeds per capitula without considering the overall seed production per plant.

Similarly, the study of Sober and Ramula (2013) found no significant relationship between seed mass and seed numbers in the invasive species *Lupinus polyphyllus* Lindl. (Fabaceae) [26]. Nevertheless, this coordination trait strategy confers advantages to *C. odorata* by facilitating increased offspring production, thereby promoting its establishment and expansion. These findings suggest that *C. odorata* exhibits distinct ecological strategies following its introduction, thereby contributing to its successful invasion of the introduced regions.

## 5. Conclusions

This study provides a quantitative analysis of the seed traits of the invasive species *C. odorata* and explicitly differentiates their effects on ecological characteristics. The ecological status of the place of origin strongly influenced trait differences, except for the exception of pappus length. Moreover, some traits were influenced by climatic conditions in the original population locations. These factors contribute to substantial variation in seed traits among populations and between introduced and native ranges. Following their introduction, the introduced populations of *C. odorata* shifted towards producing lighter seeds with enhanced dispersal ability while experiencing reduced germination rates as an associated cost. For invasive species, such strategies facilitate expansion into new environments that lack natural specialist enemies or co-evolutionary competitors.

## Figures and Tables

**Table 1:** Mixed model analysis of seed and seedling traits differences between native and invasive populations of *Chromolaena odorata.* The first column of the table shows the model structure (fixed effect) of the maximal models. TS, temperature seasonality; PD, precipitation of driest quarter. Range × Climate means interaction between range and climatic variables. Parameter estimates and significance levels of selected in the minimal model. *, **, and *** indicate *p* < 0.05, *p* < 0.01, and *p* < 0.001.

	Germination Rate (%)	Seed Mass (mg)	Pappus Length (mm)	Seed Numbers per Capitula	Terminal Velocity (m s^−1^)	Seedling Length (mm)
Intercept	68.41 ***	0.02 ***	0.49 ***	12.11 **	89.87 ***	35.53 ***
Range (R)	−21.31 ***	−0.005 **	−0.12	8.21 **	−17.56 ***	−10.03 *
PD			−0.001			
TS				0.02 *		
R*Climate			0.0001 *			

**Table 2:** Geographical location and elevation of 12 native and 10 introduced *Chromolaena odorata* populations used in the present study.

Code	Country/Region	Latitude	Longitude	Elevation (m asl)
Invasive populations				
BK	Thailand	14°25′ N	101°23′ E	739
JD	Yunnan, China	24°17′ N	100°50′ E	1263
ML	Yunnan, China	21°56′ N	101°15′ E	544
MY	Melaka, Malaysia	2°22′ N	102°21′ E	50
PH	Iligan, Philippines	8°10′ N	124°10′ E	107
SL	Kegalle, Sri Lanka	7°11′ N	80°25′ E	451
SM	Yunnan, China	22°46′ N	100°56′ E	1380
SY	Hainan, China	18°19′ N	109°12′ E	23
WX	Vientiane, Laos	17°58′ N	102°37′ E	170
YNS	Southern Vietnam	11°20′ N	107°24′ E	125
Native populations				
MCD	Tamaulipas, Mexico	23°40′ N	99°11′ W	600
MCY	Chiapas, Mexico	16°44′ N	93°09′ W	640
CUB	Pinar del Rio, Cuba	22°45′ N	82°50′ W	565
FAK	Collier, Florida, USA	25°52′ N	80°29′ W	1324
FBRO	Broward, Florida, USA	26°08′ N	80°06′ W	3
FMAR	Martin, Florida, USA	27°06′ N	80°15′ W	3
FMD	Miami, Florida, USA	25°38′ N	80°20′ W	3
MIC	Michoacan, Mexico	18°51′ N	103°37′ W	950
PM	Manati, Puerto Rico	18°12′ N	67°06′ W	103
PP	Ponce, Puerto Rico	18°12′ N	67°06′ W	103
T1	Mamoral, Trinidad	10°27′ N	61°17′ W	63
T2	Felicity, Trinidad	10°31′ N	61°25′ W	10

## Data Availability

All data generated or analyzed during this study are included in this published article (Supplementary Files) and also available from the corresponding author on reasonable request.

## Supplementary Materials

The following supporting information can be downloaded at: https://www.mdpi.com/article/10.3390/plants13131747/s1, Table S1. Climatic variables of all sampling populations; Table S2. Overall variation of the seed traits of *Chromolaena odorata*; Table S3. Seed traits (mean ± SD) of *Chromolaena odorata* from the introduced and native ranges; Figure S1. Illustration of seed pappus length measurement for *Chromolaena odorata*: measured as the length of the longest hair of pappus; Figure S2. Variance partitioning of seed traits of *Chromolaena odorata* across three levels of organization.
