# Supplementary material for: Variation in Seed Morphological Traits Affects the Dispersal Strategies of Chromolaena odorata Following Invasion
Source: Plants (Basel). 2024 Jun 24;13(13):1747. doi: 10.3390/plants13131747 (PMC11244504; doi:10.3390/plants13131747)

**Table S1.** Climatic variables of all sampling populations

| Range | Population | Precipitation of driest quarter (mm) | Temperature seasonality (standard deviation *100) |
|---|---|---|---|
| Introduced | BK | 28.00 | 186.4 |
| | JD | 52.00 | 407.3 |
| | ML | 75.00 | 351.6 |
| | MY | 332.00 | 44.0 |
| | PH | 370.00 | 91.5 |
| | SL | 384.00 | 72.3 |
| | SM | 56.00 | 364.9 |
| | SY | 75.00 | 297.5 |
| | WX | 24.00 | 253.3 |
| | YNS | 67.00 | 109.7 |
| Native | CUB | 169.00 | 218 |
| | FAK | 149.00 | 338.4 |
| | FBRO | 200.00 | 333.6 |
| | FMAR | 197.00 | 373.6 |
| | FMD | 138.00 | 315.6 |
| | MCD | 51.00 | 422.6 |
| | MCY | 23.00 | 175.2 |
| | MIC | 4.00 | 143.9 |
| | PM | 164.00 | 128.3 |
| | PP | 164.00 | 128.3 |
| | T1 | 227.00 | 59.5 |
| | T2 | 133.00 | 57.3 |

**Table S2** Overall variation of the seed traits of *Chromolaena odorata*

| Traits | Mean (range) | Magnitude of range | CV (%) |
|---|---|---|---|
| Seed mass (mg) | 0.27 (0.13-0.60) | 0.46 | 34.05 |
| Pappus length (cm) | 0.29 (0.19-0.58) | 0.39 | 33.21 |
| Number of seeds in one flower | 28 (13-40) | 27.00 | 24.48 |
| Terminal velocity (m $s^{-1}$) | 64.3 (32.2-112.3) | 80.08 | 23.25 |
| Germination rate (%) | 37.5 (6.0-78.0) | 72.00 | 47.48 |
| Seedling length (cm) | 21.6 (3.6-69.0) | 65.35 | 55.14 |

**Table S3.** Seed traits (mean ± SD) of *Chromolaena odorata* from the introduced and native ranges.

| Range | Seed mass (mg) | Seed numbers per capitula | Pappus length (mm) | Germination rate (%) | Seedling length (mm) | Terminal velocity (m $s^{-1}$) |
|---|---|---|---|---|---|---|
| Introduced | 0.22 ± 0.03 | 33 ± 3 | 0.29 ± 0.09 | 26.05 ± 10.96 | 16.50 ± 7.94 | 54.76 ± 8.05 |
| Native | 0.32 ± 0.10 | 25 ± 7 | 0.29 ± 0.10 | 47.11± 16.7 | 25.77 ± 13.00 | 72.32 ± 14.73 |

**Figure S1.** Illustration of seed pappus length measurement for *Chromolaena odorata*: measured as the length of the longest hair of pappus.

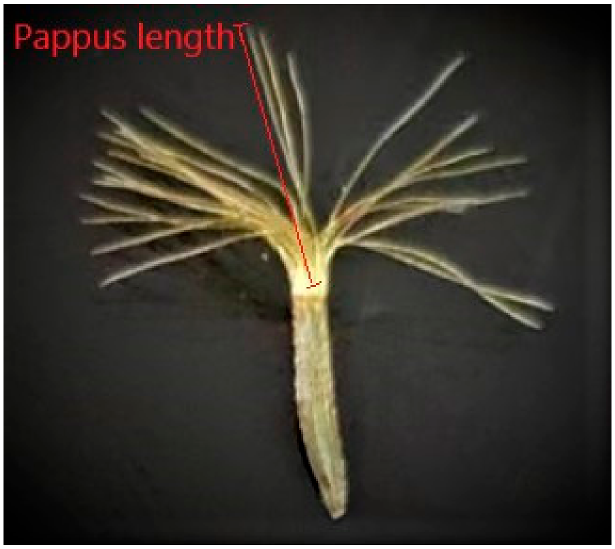

**Figure S2.** Variance partitioning of seed traits of *Chromolaena odorata* across three levels of organization.

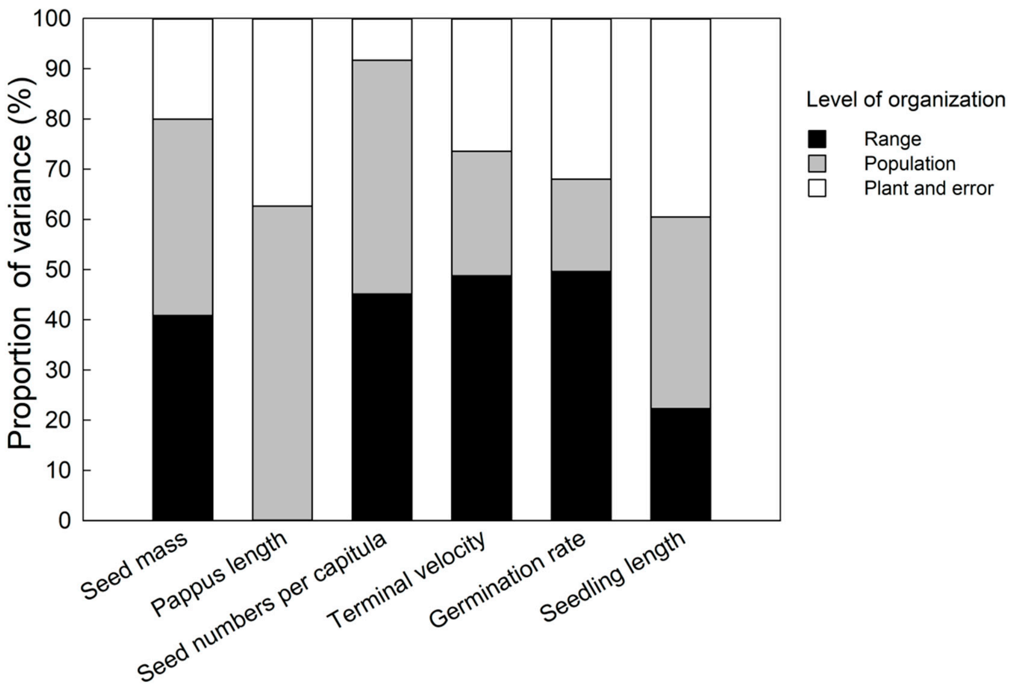

Supplement: Supplementary file 1 [file plants-13-01747-s001.zip › plants-3030931-supplementary.pdf]
